# High-throughput analysis of amino acids in plant materials by single quadrupole mass spectrometry

**DOI:** 10.1186/s13007-018-0277-8

**Published:** 2018-01-19

**Authors:** Rasmus Dahl-Lassen, Jan van Hecke, Henning Jørgensen, Christian Bukh, Birgit Andersen, Jan K. Schjoerring

**Affiliations:** Department of Plant and Environmental Sciences, Faculty of Science, University of Copenhagen, Thorvaldsensvej 40, 1871 Frederiksberg C, Denmark

**Keywords:** Amino acid analysis, Biomass, Biorefinery, Green leaves, Mass spectrometry, Protein extraction

## Abstract

**Background:**

The amino acid profile of plants is an important parameter in assessments of their growth potential, resource-use efficiency and/or quality as food and feed. Screening studies may involve large number of samples but the classical amino acid analysis is limited by the fact that it is very time consuming with typical chromatographic run times of 70 min or more.

**Results:**

We have here developed a high-throughput method for analysis of amino acid profiles in plant materials. The method combines classical protein hydrolysis and derivatization with fast separation by UHPLC and detection by a single quadrupole (QDa) mass spectrometer. The chromatographic run time is reduced to 10 min and the precision, accuracy and sensitivity of the method are in line with other recent methods utilizing advanced and more expensive mass spectrometers. The sensitivity of the method is at least a factor 10 better than that of methods relying on detection by fluorescence or UV. It is possible to downscale sample size to 20 mg without compromising reproducibility, which makes the method ideal for analysis of very small sample amounts.

**Conclusion:**

The developed method allows high-throughput analysis of amino acid profiles in plant materials. The analysis is robust and accurate as well as compatible with both free amino acids and protein hydrolysates. The QDa detector offers high sensitivity and accuracy, while at the same time being relatively simple to operate and cheap to purchase, thus significantly reducing the overall analytical costs compared to methods based on more advanced mass spectrometers.

**Electronic supplementary material:**

The online version of this article (10.1186/s13007-018-0277-8) contains supplementary material, which is available to authorized users.

## Background

A large number of research questions in basic plant biology and plant breeding require analysis of the amino acid profiles of proteins in single plant tissues or whole plants. This is required in order to improve parameters associated with plant growth, tolerance towards environmental stress and resource-use efficiency. The amino acid composition is also a key parameter in assessment of the nutritional quality of proteins in food and feed materials. Here, not only information about the composition of essential amino acids is required but also that of non-essential amino acids as a proper balance is required to improve the utilization of the proteins in the diet [1]. The current societal focus on biorefining and bio-based economy has prompted a booming interest and spurred new research activities on how to exploit proteins extracted from green biomass [2–4]. However, these studies have often been performed without assessing the nutritional values of the obtained proteins by amino acid analysis [5, 6]. Plant-derived proteins are believed to have an enormous untapped potential for animal feed, food, food ingredients and as raw materials for other valuable products in future biorefinery contexts [7].

Analysis of the amino acid profile of plant materials has hitherto relied on very time consuming methodology based on ion exchange chromatography and involving very sensitive pH adjustments as well as chromatographic run times of well above 1 h per injection [8]. Newer and faster methods based on ESI-TOF MS [9], triple quadrupole MS [10], or orbitrap MS [11] are available. These methods have proven valuable for compound identification and other non-routine analyses of amino acids and amines [12–14]. However, they require expensive high-end mass spectrometers and highly trained analytical personnel and are thus not ideally suited for routine analysis of large sample sets. Much research on plant-derived proteins in plant, animal and food science has consequently been carried out without investigations of the amino acid profile [2, 15–19]. This emphasizes the need for a more easily accessible method for high throughput analysis.

When considering the analysis time of a single chromatographic run, it is important to remember that no method for protein hydrolysis has yet been developed in which all amino acids can reliably be liberated and analyzed in one run. Typically, three separate hydrolysis methods must be used when a full profile is required, i.e. an acidic hydrolysis with prior protection of sulphur-containing amino acids (cysteine and methionine) by oxidation, an alkali hydrolysis for tryptophan, and an acidic hydrolysis for the remaining amino acids. Therefore, any gain in analysis time will be a threefold gain. This makes the run time of the chromatography the real limitation in a high-throughput amino acid analysis.

Although attempts have been made to develop a method for the analysis of underivatized amino acids [20], most methods rely on derivatization of the amino acids. This is needed due to the fact that amino acids in general do not possess chromophores or easily ionizable functional groups. A good derivatization agent provides a quantitative and preferably fast reaction, delivering either a chromophore for UV or fluorescence detection, or an easily ionizable group for MS detection. Typical derivatization strategies include 9-fluorenylmethyl chloroformate (FMOC) [21, 22], dansyl chloride [23], *ortho*-phthalaldehyde (OPA) [24], ninhydrin [25] or aminoquinolyl-*N*-hydroxysuccinimidyl carbamate (AQC) [25]. Ninhydrin is commonly used for post-column derivatization, while AQC is used pre-column, followed by fluorescence detection after reverse phase chromatography.

A special feature of using MS detection is the ability to spike the samples with stable isotope labelled internal standards matching the amino acids to be measured. This significantly improves the reproducibility and reliability over methods that rely on a single amino acid, e.g. norvaline, as internal standard [26], or no internal standard at all. The advantage of using stable isotope labeled amino acids as internal standard is that each amino acid has a different ionization efficiency and, consequently, are ionized differently by the electrospray ion source. A single internal standard compound would not be able to compensate correctly for the differential ionization efficiency, but the problem may be eliminated when a separate, chemically identical, internal standard is applied for each measured amino acid [27]. The use of stable isotope labelled amino acids as internal standard has so far been limited to a relatively small number of compounds and studies [27] despite the fact that a significant gain in precision and robustness can be obtained with marginal extra analytical costs.

The purpose of the present study was to develop a high-throughput amino acid analysis exploiting the advantages of single quadrupole MS for detection in combination with stable isotope labelled amino acids as internal standards. Using a range of plant-derived materials, we document that the developed method is fast, robust, accurate and sensitive compared to current standard methods.

## Methods

### Materials

Analytical grade AccQ-Tag kit [containing acetonitrile, borate buffer, and 6-aminoquinolyl-N-hydroxysuccinimidyl carbamate (AQC) reagent] together with Pierce Amino Acid Standard H (2.5 mM amino acid and 1.25 mM cysteine) were obtained from Waters (Millford, MA, USA). LC–MS grade acetonitrile, formic acid, sodium hydroxide, hydrogen peroxide, sodium metabisulfite, l-glutamic acid, γ-aminobutyric acid (GABA), α-aminobutyric acid (AABA), *trans*-4-hydroxy-l-proline, l-cysteic acid monohydrate, l-methionine sulfone, l-tryptophan and Cell free ^13^C–^15^N-labeled amino acid mixture were obtained from Sigma-Aldrich (St. Louis, MO, USA). Hydrochloric acid was obtained from Merck (Darmstadt, Germany). Milli-Q water (Millipore, Billerica, MA) was used for preparation of all buffers and reagents.

The matrices used in this study were whole plant and pulp after extraction of red clover (*Trifolium pretense*), rye grass (*Lolium perenne*) and lucerne (*Medicago sativa*), whole plant *Arabidopsis thaliana* and *Brachypodium distachyon*, triticale seeds, spinach leaves (*Spinacia oleracea*) and dried dog food pellets. A certified reference material, NIST-1849a infant formula reference material, commercially available from National Institute of Standards and Technology (NIST) was also included.

### Preparation of standard and internal standard solutions

The complete amino acid (AA) standard consisted of Pierce Amino Acid standard H (final concentration of 0.5 mM) supplemented with α-aminobutyric acid (AABA) (0.625 mM), γ-aminobutyric acid (GABA), cysteic acid, methionine sulfone and hydroxyproline (0.5 mM). Furthermore, the mixture was fortified with glutamic acid (2.5 mM total concentration). This mixture was then split in aliquots of 35 µL and frozen for storage.

The internal standard solution contained all 20 amino acids labeled with all ^13^C- and ^15^N-isotopes. The received 20 mM standard mixture was diluted to 0.5 mM (user concentration) split into smaller aliquots and frozen for storage.

Stable isotope labeled methionine sulfone and cysteic acid were not commercially available and were prepared in our lab from labeled methionine and cysteine according to the procedure by Jariwala et al. [28]. In brief, a performic acid mixture was prepared by adding 9 mL of formic acid and 1 mL of hydrogen peroxide (30%). The mixture was left on ice for approximately 30–60 min. Ten milligram of both labeled cysteine and labeled methionine were added to a falcon tube. The performic acid mixture was added to the falcon tube and left for 1 h at room temperature. After the oxidation, the liquid was evaporated under nitrogen to dryness. The dried residue was used for internal standard, assuming complete conversion to methionine sulfone and cysteic acid, respectively.

### Sample preparation

All plant materials and the dog food sample were freeze dried and ground by ball milling prior to analysis. The reference material was analyzed in the received condition.

### Oxidation

When analysing the sulphur-containing amino acids, an oxidation was performed prior to hydrolysis in order to protect them from degradation during heating. The oxidation agent, performic acid, was prepared by mixing 48 µL hydrogen peroxide (30% w/w) and 432 µL formic acid per sample for analysis in an appropriate container and keeping it on ice for half an hour. 480 µL oxidation agent was added to 20 mg sample in a 10 mL head space glass vial with crimp cap. The oxidation was allowed to proceed at room temperature without cap for 1 h in fume hood with gentle shaking. After oxidation, 60 mg solid sodium metabissulfite was added to quench the reaction. The samples were left in the fume hood for a few minutes to let sulfur dioxide gas escape. After all gas had evaporated from the samples, 3 mL of hydrochloric acid (6 M with 0.1% w/v phenol) was added, the vial was sealed and placed in a preheated oven at 110 °C for 24 h. After hydrolysis, the samples were allowed to cool to handling temperature and then neutralized with 4 mL sodium hydroxide (6 M), mixed thoroughly and left to cool to handling temperature. After cooling, the sample was transferred to a falcon tube and filled to a final volume of 10 mL with water. An aliquot was filtered through a 0.45 µm, 13 mm diameter nylon filter.

### Acidic hydrolysis

Approximately 20 mg of sample was weighed into a 10 mL head space glass vial with crimp cap and 3 mL hydrochloric acid (6 M with 0.1% w/v phenol) was added. The vial was sealed and placed in a preheated oven at 110 °C for 24 h. After hydrolysis, the samples were allowed to cool to handling temperature and then neutralized with 3 mL sodium hydroxide (6 M), mixed thoroughly and left to cool to handling temperature. After cooling, an aliquot was filtered through a 0.45 µm, 13 mm diameter nylon filter.

### Derivatization with AccQ-Tag

The derivatization was based on the work by Cohen [29] and the recommendations set forth by the supplier. The AQC reagent was dissolved in 1 mL acetonitrile. Slight heating was necessary to bring all AQC reagent into solution. For calibration, a series of 10 dilutions of the standard stock solution were prepared by mixing 8, 6, 5, 4, 3, 2, 1, 0.4, 0.2, and 0.1 µL, respectively, with 1.5 µL internal standard solution and adding borate buffer to a total volume of 80 µL. 20 µL AQC solution was added to each standard, giving a total volume of 100 µL. The standard was mixed thoroughly immediately after addition of AQC solution to ensure complete reaction with amino acids and minimal byproduct formation.

For sample derivatization, a mixture of 33.5 µL borate buffer, 1.5 µL internal standard mixture and 5 µL neutralized sample hydrolysate was prepared and 10 µL AQC solution was added, giving a total volume of 50 µL and mixed thoroughly immediately after addition.

For both standards and samples, after mixing, the tubes were heated at 55 °C for 10 min.

### Chromatography

Sample analysis was performed on a Waters UPLC system with a UPLC Binary Solvent Manager and Sample Manager. Derivatized amino acids were detected on a Waters QDa single quadrupole mass detector in positive mode. Separation was performed on a Cortecs UPLC C18 (1.6 µm particle size, 2.1 × 150 mm) column with a VanGuard Cortecs UPLC C18 (1.6 µm particle size, 2.1 × 5 mm) guard column. The column temperature was maintained at 55 °C. The volume injected on the column was 1 µL. Gradient elution was performed using 0.5% formic acid in water as eluent A and 0.5% formic acid in acetonitrile as eluent B. The flow rate was kept constant at 0.500 mL min^−1^ with the following gradient (expressed as solvent B): Initial conditions: 0.0% B, 0.0–0.54 min: 0.1% B, 0.54–4.00 min: 6.0% B, 4.00–4.50 min: 13.0% B, 4.50–7.50 min: 16.0% B, 7.50–8.04 min: 59.6% B, 8.04–8.05 min: 90.0% B, 8.05–8.64 min: 90.0% B, 8.64–8.73 min: 0.0% B, 8.73–10.00 min: 0.0% B.

### Detection parameters

The optimal detection parameters on the QDa are listed in Table 1, including the *m*/*z* ratio after derivatization and the voltage used to steer ions through the focusing cone in the ion source, called cone voltage.

**Table 1:** Detection parameters. Amino acids listed with their corresponding mass after derivatization (*m*/*z*) and cone voltage

Amino acid	Mass (*m*/*z*)	Cone voltage (V)
Lysine^a^	244.20	12
^13^C–^15^N–Lysine^a^	248.20	12
Glycine	245.90	15
^13^C–^15^N–Glycine	248.90	15
Alanine	260.10	16
^13^C–^15^N–Alanine	264.10	16
Serine	276.10	15
^13^C–^15^N–Serine	280.10	15
Proline	286.10	15
^13^C–^15^N–Proline	292.10	15
Valine	288.10	16
^13^C–^15^N–Valine	294.10	16
Threonine	290.10	12
^13^C–^15^N–Threonine	295.10	12
Isoleucine/Leucine/Hydroxyproline^b^	302.10	17
^13^C–^15^N–Isoleucine/^13^C–^15^N–leucine	309.10	17
Aspartic acid	304.00	15
^13^C–^15^N–Aspartic acid	309.10	17
Glutamic acid	318.00	15
^13^C–^15^N–Glutamic acid	324.00	15
Histidine	326.10	12
^13^C–^15^N–Histidine	335.10	12
Phenylalanine	336.10	16
^13^C–^15^N–Phenylalanine	346.10	16
Arginine	345.10	15
^13^C–^15^N–Arginine	355.10	15
Tyrosine	352.10	15
^13^C–^15^N–Tyrosine	362.10	15

^a^Lysine is reacted with AQC reagent twice to give two ionizable groups

^b^No internal standard available for hydroxyproline

### Calculation of resolution

The resolution was calculated based on the European Pharmacopoeia (EP) standard using the following equation:

$$R = \frac{2 \times \Delta t}{{\left( {w_{1} + w_{2} } \right)}}$$

where Δt is the difference in time of the two peaks in question and w is the width of the two peaks at half maximum height. A resolution higher than 1.2 is considered sufficient for quantification and a resolution of 2 and above corresponds to complete baseline separation.

## Results

### Specificity

**Fig. 1:**
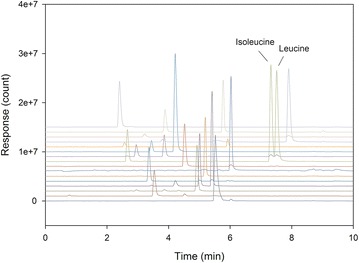
Chromatogram of the standard amino acid solution showing an overlay of all mass channels. Differently coloured lines indicate different mass channels. All peaks had a corresponding stable isotope internal standard peak at the same retention time (not shown). The only exception was hydroxyproline for which no internal standard is available. Retention times of various amino acids are listed in Table 2

The specificity of the new method was tested by injection of a standard solution of amino acids. The obtained chromatograms were overlaid and the baseline shifted upwards for illustrative purposes (Fig. 1). As evident from Fig. 1, the individual amino acids were detected at different masses, giving rise to individual peaks at separate mass channels. The only exception was the isobaric amino acids isoleucine/leucine, which appeared in the same channel and had a retention of about 7.5 min (Fig. 1; Table 1). However, the calculated resolution (EP) for this critical pair of amino acids was 1.5, i.e. sufficient for quantification.

Blank samples, consisting of a vial without sample but treated as a normal sample and going through all the analytical preparations, were injected to verify that no compounds were contaminating or co-eluting with the amino acids and thereby interfering with the ability to accurately measure peak area. As expected, no contaminating or co-eluting compounds were found on any mass channel for any peak.

### Calibration and sensitivity

Calibration curves were tested with and without internal standard to determine the effect of the internal standard on the linearity and reproducibility of the method. For calibration curves without internal standard, quadratic regression gave the best fit. Typical correlation coefficients for calibration curves without internal standard were r^2^ = 0.85–0.95 depending on the specific amino acid (data not shown). For calibration curves with the use of internal standard, linear regression gave the best fit and with very good correlation coefficients (r^2^ > 0.99), although slightly lower for histidine (see Table 2 for full list). Hydroxyproline was detected without internal standard as ^13^C–^15^N-labeled hydroxyproline was not available. Despite this, the correlation was still strong (r^2^ = 0.9922) using a quadratic regression.

**Table 2:** Retention times, correlations and sensitivity

Amino acid	Retention time (min)	Injection range (µM)	Correlation coefficient	LOD (µM)	LOQ (µM)
Alanine	5.02	0.5–40	0.9986	0.07	0.24
Arginine	3.29	0.5–40	0.9990	0.60	1.99
Glycine	3.67	0.5–40	0.9995	0.07	0.22
Histidine	2.60	0.5–40	0.9872	0.13	0.42
Hydroxyproline	2.71	0.5–40	0.9922	0.03	0.11
Isoleucine	7.35	0.5–40	0.9994	0.03	0.08
Leucine	7.60	0.5–40	0.9994	0.03	0.10
Lysine	5.60	0.5–40	0.9985	0.04	0.14
Phenylalanine	7.95	0.5–40	0.9995	0.05	0.15
Proline	5.21	0.5–40	0.9994	0.12	0.41
Serine	3.44	0.5–40	0.9995	0.20	0.66
Threonine	4.59	0.5–40	0.9994	0.07	0.22
Tyrosine	5.83	0.5–40	0.9992	0.02	0.08
Valine	6.05	0.5–40	0.9995	0.46	1.55
Aspartic acid	3.91	0.5–40	0.9989	0.11	0.36
Glutamic acid	4.29	2.5–200	0.9985	0.08	0.26
Cysteic acid	2.86	0.5–40	0.9996	0.58	1.94
Methionine sulfone	4.02	0.5–40	0.9997	0.05	0.18

All measured amino acids with their retention times, normal concentration range, correlation coefficient, limit of detection (LOD), limit of quantification (LOQ), and injection repeatability

Several optimization steps were conducted to improve performance of the QDa detector. The cone voltage was optimized for each amino acid separately, and the applied cone voltages are listed in Table 1. The effect of the formic acid concentration (0.1–1%) in the mobile phases was also tested. The best detector response and peak shape were obtained using 0.5% formic acid (data not shown).

With the chosen setup, the working range was between 0.5 and 40 µM for most amino acids except glutamic acid for which the working range was 2.5–200 µM. This working range was suitable for analyzing most plant materials without having to further dilute the samples after the hydrolysis step, thus decreasing the analytical preparation time. All correlations were strong, ranging from r^2^ = 0.9872 to 0.9997 (Table 2).

### Precision and accuracy

The injection repeatability was tested by ten consecutive injections of the same sample and found to be in the range of 0.8–3.7% (Table 3). The derivatization repeatability was tested by derivatizing the same sample five times. The repeatability ranged from 0.6 to 5.5% (Table 3). As calculated from the results in Table 3, the injection had an average contribution to the method relative standard deviation of 1.5%. The average relative standard deviation for the derivatization repeatability was 2.6% and the difference was statistically significant (*p* = 0.05) from the relative standard deviation of the injections. This shows that the derivatization step contributed significantly to the total uncertainty, even with the internal standards added.

**Table 3:** Average relative standard deviations of individual amino acid concentrations in a sample injected 10 times (injection repeatability), in 5 derivatizations from the same hydrolysate (derivatization repeatability), or in 12 different matrices analyzed three times each day for 3 days

Amino acid	Injection repeatability (%) (n = 10)	Derivatization repeatability (%) (n = 5)	Average reproducibility (%)
Alanine	1.4	3.0	3.8
Arginine	2.3	1.8	4.9
Glycine	1.1	0.8	3.9
Histidine	2.2	2.7	7.7
Hydroxyproline	3.3	4.0	18
Isoleucine	0.8	1.4	4.1
Leucine	1.0	2.9	4.6
Lysine	3.7	3.2	5.3
Phenylalanine	0.9	2.4	5.5
Proline	0.8	4.2	5.6
Serine	1.2	0.7	4.0
Threonine	1.1	1.7	4.1
Tyrosine	1.1	5.5	5.1
Valine	0.9	2.9	4.5
Aspartic acid	1.1	2.2	3.6
Glutamic acid	0.9	2.9	4.0
Cysteic acid	N.D.	N.D.	6.0
Methionine sulfone	N.D.	N.D.	5.7

*N.D.* not determined

The reproducibility for each amino acid given as relative standard deviation is given in Table 3. Rather than just testing this with well-defined standard solutions, we used samples with a complex sample matrix to give a more true picture of the expected performance of the method. The 12 different matrices were selected to cover a wide range of plant matrices as well as a commercially available dog food and a certified reference material from NIST. The relative standard deviation was on average 5.3%, and with the exception of hydroxyproline, the values ranged from 3.6 to 7.7%. A full list of relative standard deviations for all amino acids in all 12 matrices is included as Additional file 1: Table S1.

**Fig. 2:**
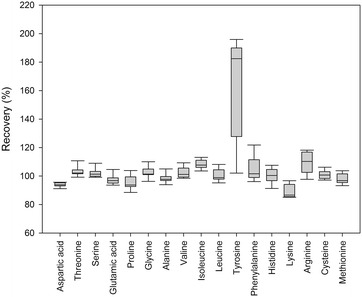
Recoveries of amino acids measure in eight different biological materials. Box plot of measured amino acid concentrations of eight different sample materials relative to corresponding values obtained from an external certified laboratory or certified values of the reference material. Box edges denotes first and third quartile. Line in box denotes median value. Whiskers denote highest and lowest values

**Fig. 3:**
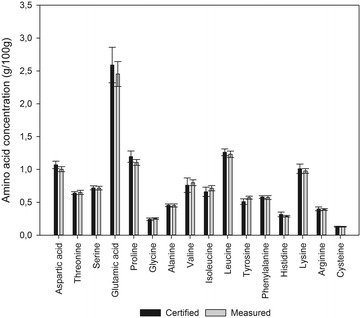
Measured amino acid concentrations compared with corresponding certified values for NIST 1849a. A comparison of the measured values (n = 9) and the certified values of the reference material NIST 1849a. Error bars denote standard deviation

The accuracy of the developed method was tested by analysing eight biological materials (Fig. 2). Recoveries were calculated relative to values obtained for the same samples analyzed by an external contract laboratory. The chosen laboratory is certified according to ISO 17025 and the used method, a modified version of ISO 13903, is accredited with the Danish accreditation authorities (DANAK). Therefore, the values obtained for the 8 different materials from this laboratory are considered reliable as the basis of a comparison. The recoveries of all amino acids except tyrosine were close to 100%, this showing very good accuracy (Fig. 2). The discrepancy for tyrosine is discussed further below. The accuracy was further documented by analysis of a certified reference material from NIST (Fig. 3). Compared to the NIST values, the recoveries ranged from 91% for histidine to 112% for isoleucine.

### Sample amount

**Fig. 4:**
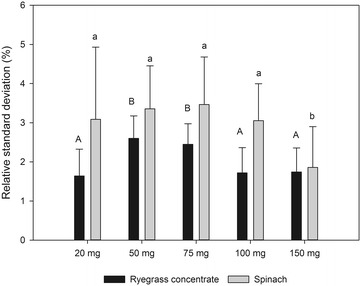
Average relative standard deviation of amino acid concentrations (n = 5) analyzed in increasing quantities of two different plant matrixes, viz. spinach (shaded columns) and a protein concentrate of ryegrass (black columns). Different letters indicate significant statistical difference based on ANOVA (*p* ≤ 0.05)

The results shown above were all obtained using a small sample amount (20 mg), which makes the developed method suitable for analyzing e.g. specific tissue samples where little material is available. To test if increasing sample quantity influenced the performance of the method, two different sample matrixes (a ryegrass protein concentrate and spinach leaves), ground to a fine powder, were analyzed in increasing quantities up to 150 mg (Fig. 4). The observed differences in relative standard deviation between the different sample quantities did not exceed 1% and was thereby almost negligible when compared to the total uncertainty of the method (approximately 5%). The developed method can therefore be applied to a range of sample amounts, also including very small samples.

## Discussion

Analysis of the amino acid composition of proteins involves several steps including protein hydrolysis, chromatographic separation and detection. So far, no protein hydrolysis method has been developed that releases all proteinogenic amino acids quantitatively in one step. In total, three separate hydrolyses are required to quantify all proteinogenic amino acids in a sample. Even though each hydrolysis may take 24 h, they can easily be performed for several hundreds of samples simultaneously, and therefore the true limitation to the throughput of the amino acid analysis is the time of the chromatographic run.

The use of a UPLC system with a Cortecs UPLC C18 (1.6 µm particle size, 2.1 × 150 mm) column allowed for a total run time of only 10 min including equilibration (Fig. 1). The last compound, phenylalanine, was eluted at around 8 min and thereafter a peak from the dimerized AQC reagent eluted in the brief column washing. When comparing to the classical ion exchange chromatography methods used for amino acid analysis, where the analytical run time required is at least 70 min [26], the method presented here is much faster and suitable for high-throughput purposes. An additional advantage of the developed method is that it allows quantification without the need for further dilutions after the hydrolysis, minimizing the analytical sample preparation time and risk of introducing error.

Using mass spectrometry for detection has the advantage that the separation of non-isobaric compounds (compounds of different masses) becomes less critical. This benefit was also demonstrated in the present work, resulting in successful quantification of the individual amino acids despite the fact that the chromatographic separation overall was not very good (Fig. 1). The only critical chromatographic separation in the developed method, as has also commonly been observed in different column systems [19, 30, 31], was the isoleucine/leucine eluting at around 7.5 min. The resolution for this critical pair was calculated to be 1.5 (EP), meaning that baseline separation was almost achieved. The calculated resolution was well above the 1.2 that is considered sufficient for quantification and therefore deemed acceptable.

The detection limit of 0.03–0.60 µM for the method developed here was in line with that obtained using high-end mass spectrometers [32] and was approximately one order of magnitude better than fluorescence detection with the same derivatization chemistry [29]. The reproducibility was typical for this type of method [19, 22]. Hydroxyproline was an exception, showing a poor reproducibility (18%). This can in part be explained by very low concentrations of hydroxyproline present in the plant material. The largest contributor to the increased uncertainty was most likely the absence of an internal standard for hydroxyproline. This will result in differences in ionization efficiency from sample to sample and therefore increase the uncertainty of the quantification (see also [27]). By addition of an isotope labelled internal standard for each measured amino acid, the problem of small ionization efficiency fluctuations can be eliminated. Isotope labelled amino acids for hydrolysed samples are commercially available, but their use as internal standards has been limited to a relatively small number of compounds and studies [27, 33, 34]. To the best of our knowledge, stable isotopes of the oxidized sulphur-containing amino acids methionine sulfone and cysteic acid, which are present in oxidized samples, have never before been used as internal standards. They can easily be synthesized [28] and we have in this study successfully used them as internal standards to significantly improve the quantification of these two amino acids.

The measured values for the NIST 1849a reference material was in good agreement with the certified value for all amino acids. The recovery was also evaluated by comparing values obtained from six plant derived materials and a pet food sample with values from analysis of the exact same samples by an external laboratory. In general, good agreement, i.e. recovery close to 100%, was found for most amino acids (Fig. 2). Our method slightly underestimated lysine and aspartic acid, while isoleucine was slightly overestimated. All three compounds were, however, within the certified uncertainty when comparing to NIST 1849a (Fig. 3), when the combined uncertainty of our and the NIST analysis is taken into account [35].Furthermore, for tyrosine the recovery was significantly higher for all matrices except the dog food and NIST 1849a (comparison with certified value). To prevent halogenation of tyrosine during acidic hydrolysis, phenol was added [36]. In the hydrolysis performed in our study, a phenol concentration of 0.1% (w/v) was used, as prescribed in the official ISO 13903 Standard [26]. Based on the information obtained from the contract laboratory, they used a significantly lower concentration of phenol, which will most likely explain the lower values obtained with their method. The difference was larger for plant samples than for NIST 1849a and dog food. This indicates that the used plant materials promote halogenation, influencing the results found for tyrosine at the contract lab. The results point out that for plant derived samples the recommended 0.1% phenol has to be used.

The speed, sensitivity, precision and accuracy of the method presented here is equal to methods based on high-end mass spectrometers [12]. Along with this, our method offers advantages of relatively low instrument price and personnel costs, thus providing an attractive tool for high-throughput analyses of amino acid profiles in plants, feed and food materials.

## Conclusion

Combining UHPLC and mass spectrometry enables amino acids to be analysed significantly faster than by older methods using UV or fluorescent detection. By exploiting the specificity of the mass detection, the typical cycle time (time from injection to injection) can be reduced to approximately 11.5 min, making the method ideal for high-throughput analysis. The use of a single quadrupole mass spectrometer make the analysis a cheaper alternative to other recently published methods relying on triple quadrupole technology or high-resolution mass spectrometers without compromising the sensitivity and reproducibility. The method is compatible with hydrolyzed and oxidized samples for protein determination and is suitable for plant materials due to its high sensitivity. The analysis is also compatible with free amino acid determination and more amino acids, such as α- and γ-aminobutyric acid, can be added to the analysis.

## Additional file

**Additional file 1. Table S1.** Overview of relative standard deviations (%) separated by amino acid and sample matrix for analyses over 3 days (n = 9).
